# The Canine Morbillivirus Strain Associated with An Epizootic in Caspian Seals Provides New Insights into the Evolutionary History of this Virus

**DOI:** 10.3390/v11100894

**Published:** 2019-09-25

**Authors:** Wendy K. Jo, Martin Peters, Aidyn Kydyrmanov, Marco W. G. van de Bildt, Thijs Kuiken, Albert Osterhaus, Martin Ludlow

**Affiliations:** 1 Research Center for Emerging Infections and Zoonoses, University of Veterinary Medicine Hannover, 30559 Hannover, Germany; wendy.karen.j.lei@tiho-hannover.de (W.K.J.); Albert.Osterhaus@tiho-hannover.de (A.O.); 2 Chemisches und Veterinäruntersuchungsamt Westfalen, 59821 Arnsberg, Germany; Martin.Peters@cvua-westfalen.de; 3 Laboratory of Viral Ecology, Institute of Microbiology and Virology, 050010 Almaty, Kazakhstan; kydyrmanov@yandex.kz; 4 Department of Viroscience, Erasmus Medical Center, 3000 CA Rotterdam, The Netherlands; m.vandebildt@erasmusmc.nl (M.W.G.v.d.B.); t.kuiken@erasmusmc.nl (T.K.)

**Keywords:** morbillivirus, canine distemper virus, epizootic, Caspian Sea, Lake Baikal, *Pusa sibirica*, Pusa capsica

## Abstract

Canine morbillivirus (canine distemper virus; CDV) is a worldwide distributed morbillivirus that causes sporadic cases and recurrent epizootics among an increasing number of wild, feral, and domestic animal species. We investigated the evolutionary history of CDV strains involved in the 1988 Lake Baikal (CDVPS88) and the 2000 Caspian Sea (CDVPC00) seal die-offs by recovery of full-length sequences from archived material using next-generation sequencing. Bayesian phylogenetic analyses indicated that CDVPC00 constitutes a novel strain in a separate clade (tentatively termed “Caspian”) from the America-1 clade, which is comprised of older vaccine strains. The America-1/Caspian monophyletic group is positioned most basally with respect to other clades and is estimated to have separated from other CDV clades around 1832. Our results indicate that CDVPC00 recovered from the epizootic in the Caspian Sea in 2000 belongs to a previously undetected novel clade and constitutes the most ancestral wild-type CDV clade.

## 1. Introduction

Canine morbillivirus (canine distemper virus; CDV) is a member of the genus *Morbillivirus*, subfamily *Orthoparamyxovirinae*, within the family *Paramyxoviridae*, and contains a negative-sense single-stranded RNA genome of 15,690 bp. This virus has a broad host range and is transmitted within and between multiple carnivore species of the order Carnivora, especially members of the families Canidae, Felidae, Mustelidae, and Procyonidae, with individual cases and more sporadic epizootics documented in the families Ursidae, Phocidae, Viverridae, and Hyaenidae [1]. The true host range of CDV remains to be determined, as large outbreaks of canine distemper have occurred in colonies of rhesus macaques (*Macaca mulatta*, order Primates) in China [2,3] and cynomolgus macaques (*Macaca fascicularis*, order Primates) in Japan [4], a few cases have been recorded in collared peccaries (*Tayassu tajacu*, order Artiodactyla) in the USA [5] and captive marmots (*Marmota caudata*, order Rodentia) in Switzerland [6], and an individual case has been observed in a collared anteater (*Tamandua tetradactyla*, order Pilosa) in Brazil [7].

CDV has a global distribution and is classified into the following major clades: America-1 (vaccine strains) and -2, Asia-1 and -2, Africa-1 and -2, Europe-1/SouthAmerica-1, European wildlife, and Arctic [8]. However, newer clades have been identified in recent years, including America-3, -4, and -5 [9,10,11], SouthAmerica-2 and -3 [12,13], and Asia-3, -4, and -5 [14,15,16]. Additional clades will undoubtedly be discovered as global surveillance of CDV improves, given the paucity of available sequences from central Asia and many regions of Africa. This classification has largely been based on the analysis of complete open reading frames of the hemagglutinin (H) glycoprotein [17] and, to a lesser extent, on partial sequences of phosphoprotein (P) [18] and of the signal peptide region of the fusion (F) glycoprotein [19]. However, the increasing availability of whole-genome sequences derived from tissue samples or viruses isolated from Vero-canine CD150 cells will facilitate improved phylogenetic analyses and the identification of molecular determinants underlying CDV cross-species infection.

Canine distemper is variable among different species with respect to levels of morbidity and mortality, but the basic pathogenesis of this disease is consistent. Intra- or interspecies transmission of CDV occurs via multiple routes including aerosols, fomites, and infected bodily fluids such as urine or saliva and possibly by transmission through predation [20]. The early events of CDV infection are assumed to be analogous to those of measles virus (MV) infection in primates, in which alveolar macrophages or dendritic cells are initially infected [21] prior to virus spread to local lymph nodes in which the infection is greatly amplified in activated B- and T-lymphocytes [22]. CDV infection of immune cell populations is mediated by the cellular receptor CD150 (SLAMF1) [23]. A viremic phase of infection results in additional virus spread to lymphoid tissue throughout the body and the induction of generalized immunosuppression [24]. In the latter stages of the infection, CDV uses Nectin-4 (PVRL4) as a cellular receptor to infect epithelial cells throughout the body prior to shedding into the environment [22,25,26]. In a high percentage of cases, CDV also spreads to the central nervous system leading to acute or persistent neurological sequelae [27,28]. 

Mass die-offs of phocid species as a result of CDV infection were documented in epizootics occurring in Lake Baikal in 1987–1988 [29] and the Caspian Sea in 1997 and 2000 [30,31]. CDV infection resulted in the deaths of several thousand Baikal seals (*Pusa sibirica*) in 1987–1988 [29] and of approximately 7000 and 10,000 Caspian seals (*Pusa caspica*) in the Caspian Sea in 1997 and 2000 respectively [30,31]. Caspian seals are listed as endangered by the International Union for Conservation of Nature [32]. Previous sequence analysis of the F and H glycoproteins have identified the Baikal strain of CDV as a member of the Arctic clade [33]. However, little data are available on the Caspian strain of CDV, with only a short sequence (109 bp) of the P gene reported previously [34], and the limited phylogenetic analysis performed on sequences derived from 1997 and 2000 indicated that it is distinct from all known laboratory strains and field isolates of CDV [30,31,34].

In the present study, we investigated the temporal dynamics and evolutionary history of CDV strains responsible for the epizootics in Lake Baikal in 1988 and in the Caspian Sea in 2000 using recently generated whole-genome sequences from CDV-infected European wildlife to facilitate comparison with contemporary CDV strains.

## 2. Materials and Methods

### 2.1. Tissue Samples

Frozen lung and spleen samples from a CDV-infected Baikal seal (PS88) and lung and kidney samples from three Caspian seals (PC00-46, PC00-20, PC00-21) that had died during the 1988 and 2000 epizootics of canine distemper, respectively, were processed for use in this study. In addition, we analyzed three CDV-positive frozen lung and tonsil samples originating from European (North Rhine-Westphalia, Germany) free-living wild carnivores, i.e., two raccoons (*Procyon lotor*) (S460, S466) from 2015 and a fox (*Vulpes vulpes*) (S272) from 2016. All frozen tissues had been stored at −80 °C.

### 2.2. Sample Processing

Tissue samples were processed using a viral enrichment protocol as previously described [35]. Briefly, the samples were subjected to three cycles of mechanical homogenization and freeze–thawing followed by cell debris centrifugation and filtration of bacteria using 0.45 μm spin columns. RNA was extracted from the homogenates using TRIzol (Thermo Fischer Scientific), and cDNA was reverse transcribed with Superscript IV (Thermo Fischer Scientific) using non-ribosomal hexamers. Subsequently, a random PCR amplification of the nucleic acid contents in the samples was performed with Phusion polymerase (NEB). 

### 2.3. Generation of Full-Length Genome

The samples were further prepared following the Nextera XT DNA Library Prep Kit protocol (Illumina, San Diego, CA, USA) and sequenced on an Illumina MiSeq system using the MiSeq Reagent Kit v3 (600 cycles; Illumina). Reference assembly was performed with the software CLC Genomics Workbench 10. Genome ends were generated using rapid amplification of cDNA ends (RACE) as described previously [35]. For the 3′ end or leader sequence, a poly(T) adaptor was used as forward primer and an oligonucleotide with sequence 5′-ATCACCGACCAATCTAACAAGTCTATCC-3′ was used as reverse primer. For the 5′ end or trailer sequence, an oligonucleotide with sequence 5′-AGTGCACTGATTAGAAAC-3′ was used as forward primer and a poly(T) adaptor as reverse primer. 

### 2.4. Phylogenetic Analyses

Sequences generated in this study were aligned to complete genomes of CDV deposited in GenBank using MAFFT [36]. Maximum-likelihood analyses on MEGA7 [37] were performed with 1000 bootstraps and general time reversible (GTR + G + I) as best-fit model according to the Bayesian information criterion. To determine the clock-likeliness of the sequences, we used TempEst software [38]. Recombination analyses were performed with the RDP4 package [39], which included the methods RDP, GeneConv, Bootscan, MaxChi, Chimera, SiScan, and 3Seq with cut-off *p*-value of 0.01. Sequences without temporal signal and which went through extensive recombination were excluded from the Bayesian analyses. Bayesian estimations were then carried out with BEAST v2.4.6 software [40]. The sequences were partitioned into coding regions of N, P, M, F, H, and L and non-coding sequences including leader, trailer, as well as gene start and gene end of each gene. The tests were run for 50 million generations, sampling every 1000 steps with the following priors: coalescent constant population, strict clock model rate, and Hasegawa–Kishino–Yano as substitution model. In addition, maximum-likelihood analyses of the H gene were also run 20 times with 100 bootstrap replicates and GTR model using RAxML version 8.2.10 [41].

## 3. Results

### 3.1. Identification of a Novel CDV Clade in Caspian Seals

**Figure 1:**
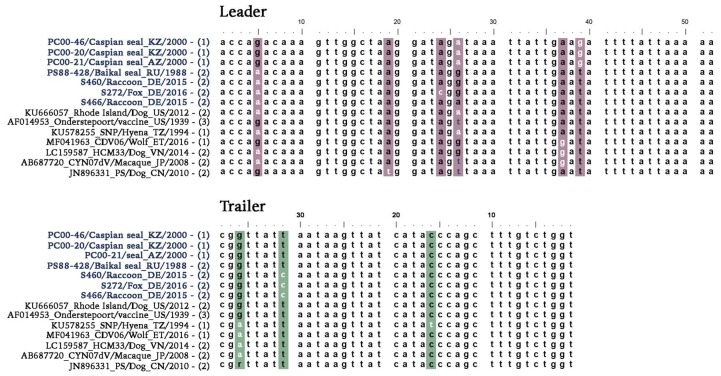
Genome termini alignment of CDV sequences. Nucleotide changes identified within the leader and trailer sequences of CDV genomes are highlighted in color. Selected sequences were generated in this study and other studies by next-generation sequencing (NGS, (1), (RACE, 2) or RNA ligation combined with PCR (3).

The evolutionary origins of CDV strains associated with two well-documented epizootics in pinniped species, Baikal seals in 1987–1988 and Caspian seals in 2000, were investigated in conjunction with the analysis of contemporary CDV strains circulating in wildlife in Germany. Full-length genome sequences of three Caspian seal CDV strain variants PC00-46, PC00-20, PC00-21 (GenBank accession nos. MN267064–66), one Baikal seal CDV strain variant PS88-428 (GenBank accession no. MN267063), two raccoon CDV strain variants S460, S466 (GenBank accession nos. MN267062, MN267060), and one fox CDV strain variant S272 (GenBank accession no. MN267061) were recovered by next-generation sequencing (NGS) and RACE from frozen archived infected tissue material (Table 1). Alignment of leader and trailer sequences from these strains with previously published CDV sequences, whose genome termini identity was confirmed using RACE or NGS, showed unexpected sequence variation (Figure 1). The CDV strains from Baikal seals and German wildlife presented a nucleotide change from A to G at genomic position 5 (g.5A>G) in the leader sequence in comparison to most other published sequences. However, this nucleotide change has also been observed previously in four other strains encompassing different species and monophyletic groups (Figure 1). This nucleotide change may be more prevalent than sequence alignments would indicate, given that the majority of CDV termini sequences were not derived de novo but instead represent primer sequences based on previously published authentic Onderstepoort strain termini sequences which are assumed to be invariable among CDV strains. 

Additionally, we identified a large number of unique amino acid changes in the Caspian seal CDV strain, a lower number in the German wildlife carnivores, and an even lower amount in the Baikal seal strain compared to 94 full-length CDV genomes (Table 2). Potential variability in SLAMF1 and Nectin-4 binding sites of the newly generated sequences was also investigated on basis of the crystal structure of the complex formed by MV-H interacting with marmoset SLAMF1 (91% similarity with human SLAMF1 [42]) and human Nectin-4 [43] (Tables S1 and S2). Most SLAMF1 and Nectin-4 interaction sites were found to be conserved. However, a unique change at position 191 was identified in strain PS880-428, which interacts with SLAMF1-Site III. Variablity at position 459, which interacts with Nectin-4-Site I, was also identified in several CDV lineages.

**Figure 2:**
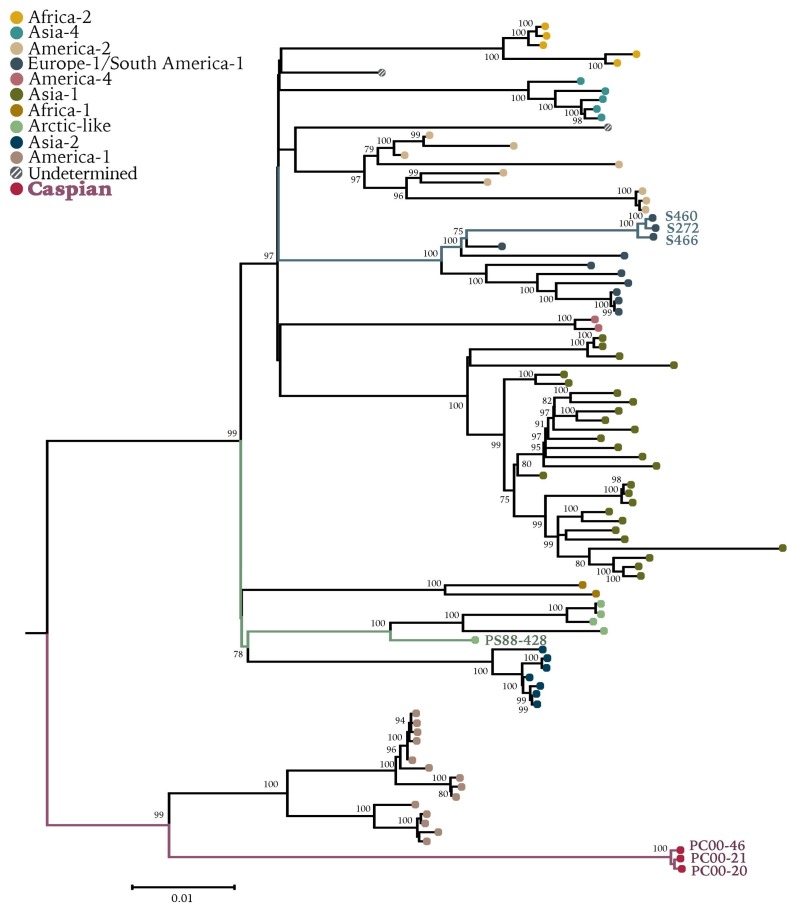
Maximum-likelihood phylogenetic tree of 94 CDV full-length genomes. Bootstrap values >70 are presented at the nodes. Phocine distemper virus (GenBank accession no. NC_028249.1) was used as outgroup. Sequences generated in this study are presented with colored branches and their respective tip name. CDV clades are presented by tip colors. Detailed information on sequences used can be found in Figure S1.

**Figure 3:**
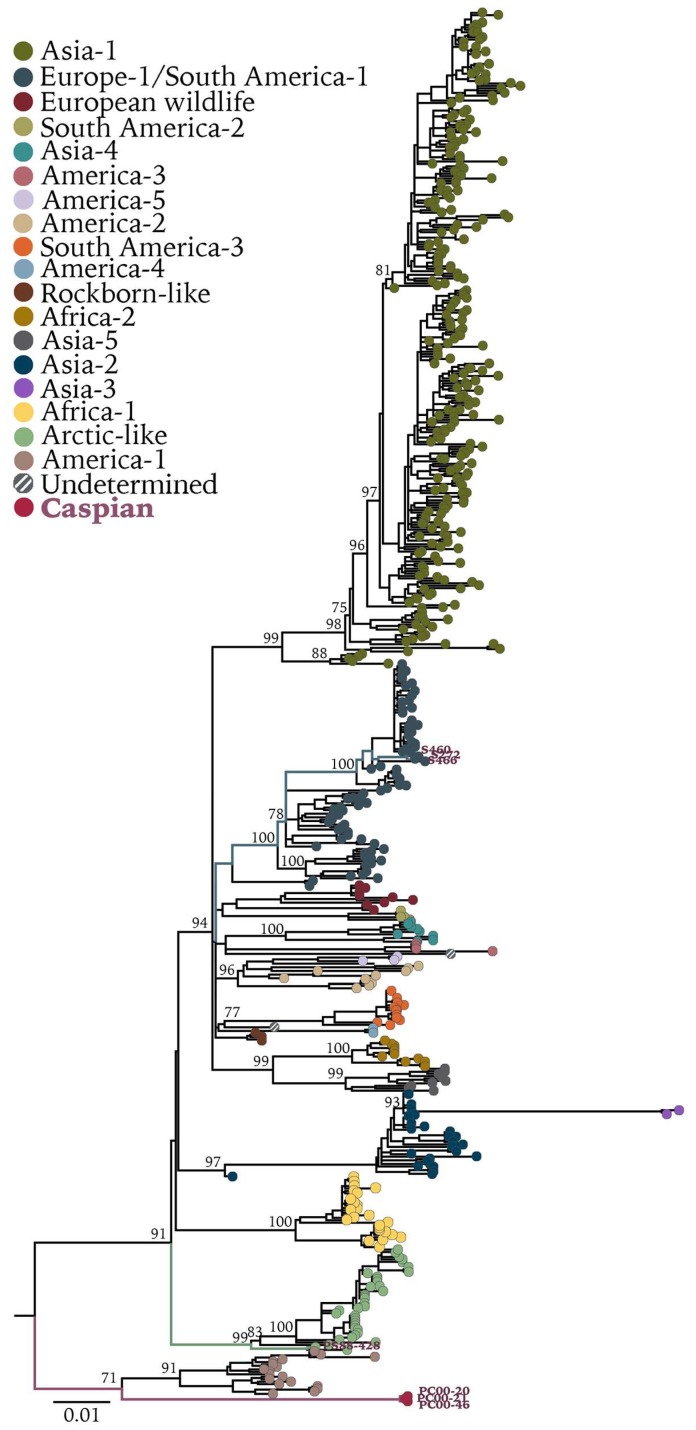
Maximum-likelihood tree of 571 complete CDV-H gene sequences. Bootstrap values >70 of major clades are presented at the nodes. H gene sequence of phocine distemper virus (GenBank accession no. NC_028249.1) was used as outgroup. Sequences generated in this study are presented with colored branches and their respective tip name. CDV clades are presented by tip colors. Detailed information on sequences used can be found in Figure S5.

Maximum-likelihood phylogenetic analyses of full-length genomes (Figure 2) and H gene sequences (Figure 3) showed that CDVPS88-428, the oldest recovered sequence of a wild-type CDV strain, grouped within the Arctic clade, as expected on the basis of previous analyses [9,23]. However, it diverged from strains within the same clade that are circulating in Siberian tigers (Panthera tigris altaica) in Russia and in domestic dogs (Canis lupus familiaris) and red foxes in Italy (Figure 2). Sequence comparison between the H protein of CDVPS88-428 and another CDV Baikal seal strain (GenBank accession no. CAA59357.1) from 1988 showed six amino acid changes: M26V, I27T, I84V, V103I, S268T, P410T. Caution must be applied with respect to ascribing these differences to natural variation, as the previously published hemagglutinin sequence was derived from a virus isolate which had been adapted to growth in Vero cells [33]. However, variation was also observed between Caspian CDV sequences at amino acid 410 (I/M). The strains circulating in German wild carnivores grouped within the Europe-1 clade (Figure 2 and Figure 3) and are related to an older strain (CDV5804) found in a domestic dog in 1989, with only minor nucleotide changes identified between raccoon and fox CDV sequences. The Caspian seal CDV strain forms its own clade, tentatively named Caspian, grouping with the America-1 clade primarily comprised of vaccine and laboratory-adapted strains (Figure 2 and Figure 3). CDVPC00 showed 89.5–93.7% sequence identity to all other CDV strains, with 80 unique amino acid changes (Table 2), making this a newly identified strain in a separate clade. Moreover, within the three recovered strain variants PC00-46, PC00-21, and PC00-21, unique genomic differences were detected. These include 16 synonymous and 3 nonsynonymous mutations when using using PC00-46 as the reference strain variant, with amino acid changes in phosphoprotein (P90L), F glycoprotein (M126T), and H glycoprotein (I410M) (Table S3). No changes in the consensus were detected between full-length genome sequences recovered from different tissues originating from the same animal. 

### 3.2. Divergence Dates between CDV Clades

For the estimation of divergence dates of CDV based on the complete genome, we excluded Snyder Hill and Onderstepoort vaccine-related strains (GenBank accession nos. EU726268, HM046486, KY971529, KY971531, KY971530, MF926604, MF926601, MF926602, and MF926603). These sequences were excluded from analysis because of their high similarity of approximately 99% at the nucleotide level to the modified live vaccine (MLV) strains Snyder Hill and Onderstepoort, despite the recent recorded date of collection. The sequence Phoca/Caspian/2007 (Genbank accession no. HM046486) was excluded from subsequent date divergence and evolutionary rate analyses because of its high similarity of 99.9% to the sequence Shuskiy collected in 1989 in the same region from a mink (GenBank accession no. HM063009). Moreover, both sequences are 99.7% identical at the nucleotide level to the vaccine strain CDV3 (Genbank accession no. EU726268.1). Two Onderstepoort sequences (GenBank accession nos. AF305419 and AF014953) were also excluded from analysis because of unclear date of origin in the literature. In addition to vaccine-related strains, we also excluded CDV strains that went through extensive genetic recombination events from downstream analyses (Table S4), as determined by the application of all detection methods in the package RDP4. Recombination can distort tree topology and influence mutational evolutionary analyses [44]. The strains excluded were the Asia-1 strains ZC (KJ994343), HLJ1 (JX681125); the Asia-2 strain 007Lm(AB474397); the Asia-4 strain 4-TH (MH496775); and America-2 strains 2645 (AY445077), 2654 (AY466011), 2646 (AY542312), 2601 (AY443350), and 2689 (AY649446).

**Figure 4:**
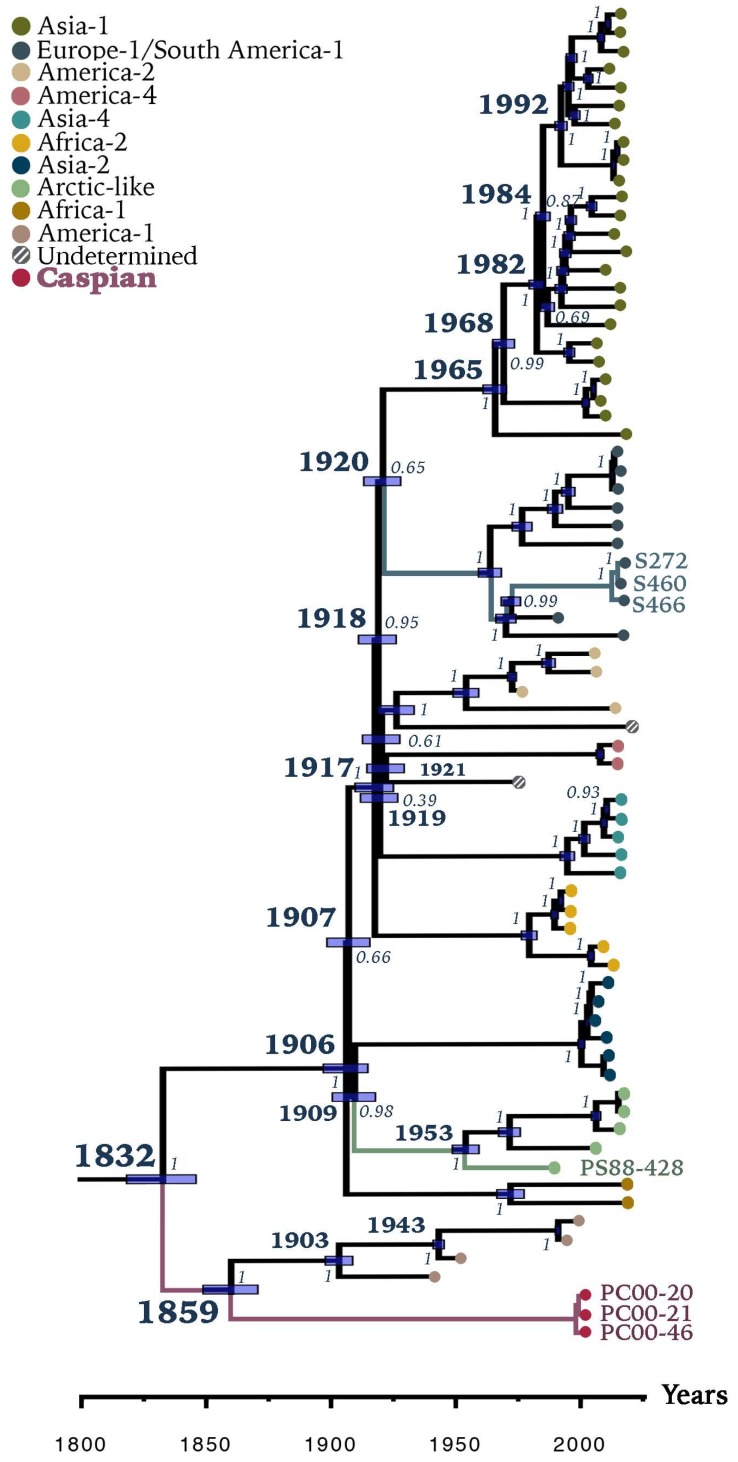
Bayesian phylogenetic tree of 73 CDV full-length genomes. Most common recent ancestor ages of major clades are presented at the nodes with posterior values in cursive. Grey horizontal bars indicate the 95% highest posterior density (HPD). Phocine distemper virus (GenBank accession no. NC_028249.1) was used as outgroup. Sequences generated in this study are presented with colored branches and their respective tip name. CDV clades are presented by tip colors. Detailed information on sequences used can be found in Figure S2.

Bayesian estimates indicated that the Caspian and America-1 monophyletic group diverged around 1832 (95% HPD:1818–1846) from all other CDV strains found to date, while shortly thereafter, the Caspian clade itself diverged from America-1 around 1859 (95% HPD:1849–1871) (Figure 4). We also performed a Bayesian phylogenetic analysis excluding the America-1 clade, because of the non-natural evolutionary history that MLVs usually undergo. Divergence dates appeared to be more recent when excluding America-1 clade (Figure S3). For instance, under this scenario, separation between the Caspian clade and all other clades appeared to have taken place around 1870. As divergence dates were significantly shifted when excluding America-1, it was necessary to include this clade in the analyses because of its basal position and close relationship with the Caspian clade. 

### 3.3. Evolutionary Rate of CDV

For the estimation of the substitution rate of CDV based on the complete genome, we excluded the same sequences that were previously taken out of the analysis for the Bayesian estimation of the divergence dates. The overall evolutionary rate of CDV was estimated to be 4.24 × 10^−4^ nucleotide substitutions per site per year (subs/site/year), The estimated substitution rates (subs/site/year) of each individual protein were: for N, 2.69 × 10^−4^ (95% HPD: 2.37–3.00 × 10^−4^), for P, 2.43 × 10^−4^ (95% HPD: 2.13–2.71 × 10^−4^), for M, 2.49 × 10^-4^ (95% HPD: 2.15–2.84 × 10^−4^), for F, 3.74 × 10^-4^ (95% HPD: 3.36–4.14×10^−4^), for H, 3.3 × 10^−4^ (95% HPD: 2.94–3.65 × 10^−4^), for L, 2.65 × 10^−4^ (95% HPD: 2.27–2.74 × 10^−4^), and for non-coding sequences, 5.95 × 10^−4^ (95% HPD: 5.33–6.56 × 10^−4^). 

Several outlier strains were identified through the estimation of the temporal signal of CDV full-length genomes (Figure S4). The analysis suggested that the Caspian strain variants PC00-46, PC00-20, and PC00-21, America-2 strains 2645, 2654, 2646, and Asia-1 strains ZC and HLJ1 were more recent than what indicated by the date given, whereas the Arctic sequence PS88-428 and America-2 sequences R252 (KF640687), 164071 (EU716337), and A75/17 (AF164967) appeared to be older than thought. In summary, the Caspian seal CDV sequences reported in this study were derived from original tissue material dating from the epizootic in 2000, with no evidence of genetic recombination and a high genetic divergence with respect to other CDV strains. 

## 4. Discussion

Outbreaks of CDV infection continue to occur in domestic animals and wildlife despite the introduction of vaccines for domestic carnivores in the mid-20th century. Diverse CDV clades circulate around the world, with increased surveillance leading to the recent discovery of new clades such as America-3 and Asia-4 [14,45]. This has been facilitated by the introduction of new technologies such as NGS which enables a more rapid assembly of full-length virus genome sequences, which are more powerful for resolving phylogenetic relationships than single genes for such analyses [46]. This also helps in the identification of recombinant CDV strains which complicate Bayesian phylogenetic analysis [14,47,48] and provides a better understanding of the evolutionary background of commonly used laboratory vaccine and “wild-type” isolates of CDV. In this study, we generated full-length genomes sequences of CDV strains associated with historical outbreaks in seal species and from German wild carnivores and report the molecular characterization of a divergent novel clade basal to all other recognized CDV clades, which was associated with the CDV epizootic in Caspian seals in 2000. This supports a more limited analysis of a single partial P gene sequence recovered from the brain of an infected Caspian seal during the 1997 epizootic [30]. 

**Figure 5:**
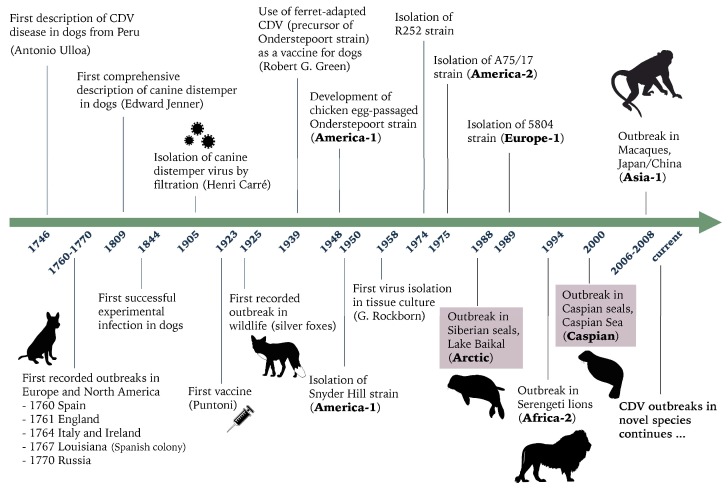
Timeline of relevant historical dates of canine distemper. Highlighted in pink are the two seal epizootics investigated in this study.

The origins of CDV (Figure 5), inferred from historical literature, are much later than those of the closely related measles virus and rinderpest virus. CDV is suspected to have originated in South America and spread to Europe in the mid-18th century, with the first description of a disease syndrome resembling canine distemper, reported to be similar to measles in dogs in Peru, made in 1746 by Antonio de Ulloa [49,50]. Shortly thereafter, the disease reached Europe with a deadly disease outbreak among dogs reported in Spain in 1760. During the 1760s, the epizootic subsequently spread to other countries including France, England, Ireland, Germany, Italy, and Russia, resulting in the deaths of thousands of dogs [49]. Although it can be assumed that wildlife was exposed to CDV throughout the late 18th and 19th century, the first report of CDV in a wildlife carnivore species was in 1925 following outbreaks of distemper in captive silver foxes (*V. vulpes*) in the USA [51]. The first attempts to control the spread of distemper was made in 1923, following the development and testing of the first experimental vaccine [52]. The subsequent introduction of more efficacious commercial CDV vaccines for domestic dogs in the mid-20th century [53] has not prevented the continual endemic transmission of CDV in wild carnivores species, as CDV continues to cause outbreaks in wildlife worldwide with occasional spillover into domestic dogs in countries with high rates of vaccination [9,54,55]. In contrast to the successful eradication campaign of rinderpest virus, the high capacity of CDV for sustained interspecies transmission means that prospects for eradication of this disease are remote. Moreover, CDV appears to have a much wider or expanding host range than was previously recognized, given the large number of new host species that have been infected in recent years including rhesus macaques, giant pandas (*Ailuropoda melanoleuca*), tigers, and seals (phocid species/pinnipeds), with associated high rates of morbidity and mortality [3,29,31,56,57].

CDV strains belonging to the Arctic clade appear to be widely dispersed geographically, as Arctic or Arctic-like strains have been detected in Greenland, Italy, North America, and Iran [55,58,59,60]. The CDV outbreak in Lake Baikal was most likely generated by a spillover from terrestrial carnivores in the vicinity. Our analysis indicates that the Baikal seal CDV strain sits most basally to all other recognized strains in the Arctic clade and has a different evolutionary pathway compared to the more recent strains found in Italy [61]. It was previously reported that the Arctic strain has become endemic in Lake Baikal as it continues to circulate among Baikal seals [62]. It would therefore be interesting to analyze and compare recent Baikal seal CDV sequences to the 1988 Baikal seal CDV sequence to identify possible nucleotide changes that might indicate adaptation to the host species and to provide an additional reference point to better assess the evolutionary rate of CDV.

Our results, which include the newly identified Caspian clade, indicate an earlier date of divergence (early-19th century) between this America-1/Caspian cluster and all other CDV strains found to date, compared to previous estimations of 1855 [14] and 1886 [63], in which the Caspian clade was absent or was based only on the hemagglutinin gene, respectively. The findings in this study support the hypothesis that recent CDV strains emerged from the America-1/Caspian clades in the early 19th century. The America-1 clade is comprised of MLVs, which were developed more than 50 years ago. The Snyder Hill and Onderstepoort strains were the first MLVs developed and have not been discovered endemically circulating in wildlife in the last half-century. More recently reported strains in the America-1 clade appear to be isolated cases representing derivatives of MLVs introduced into the wild, rather than direct wild-type decedents of the wild-type ancestor of all America-1 clade viruses. Therefore, the relationship between America-1 and Caspian clades is intriguing. Our analysis suggests that they had a common ancestor in approximately 1859, after which they separated, and the Caspian strain was possibly kept circulating in unknown reservoir species, which eventually infected the Caspian Sea pinnipeds. Given the high degree of divergence between the Onderstepoort and Caspian clades (6.35%) and the absence of many distinctive Onderstepoort specific genetic signatures, it is unlikely that the Caspian strain is an evolved MLV. The close relationship of the Baikal strain to other strains within the Arctic clade also suggests that infection of seals and subsequent intraspecies transmission do not require the accumulation of extensive mutations.

The overall evolutionary rate of CDV was estimated to be 4.24 × 10^−4^ nucleotide substitutions per site per year (subs/site/year). This rate is in a similar range to the previously calculated substitution rate of 2.46  ×  10^−4^ [14], which was estimated using a more limited number of CDV full-length sequences and did not include the Caspian clade. It is also rather similar to the estimated rate of 4.8  ×  10^−4^ determined by a study which only used the H gene [63]. However, the substitution rate calculated in this study is slower in comparison with other rates (7.41–11.35  ×  10^−4^ [8,64]) that were estimated by using only the H gene, which is under high selection pressure. The F and H glycoproteins had the fastest substitution rates, as would be expected from their role in virus–host binding and cell entry, implying that selective pressures act principally on these proteins. Genetic mutations in these proteins may be expected to contribute to virus jump and adaptation to new hosts. However, although specific amino acid positions at 530 (multiple changes) and Y549H in the H glycoprotein have been linked to interspecies transmission [65] via modulation of receptor interactions, no supportive evidence via mechanisitc in vitro assays is avaliable thus far.

## 5. Conclusions

Whereas the Baikal strain of CDV is closely related to other strains within the Arctic clade, we conclude that the Caspian clade is the most basal CDV clade recognized to date and has a different evolutionary history compared to all other CDV strains. The true reservoir, origins, and geographical distribution of this strain remain largely unknown. Enhanced CDV surveillance and molecular characterization of CDVs from wildlife species from central Asia are therefore warranted to determine if the Caspian clade is restricted to Caspian seals in the Caspian Sea or if strains from this clade are also present in terrestrial carnivores, especially in regions of Azerbaijan and Kazakhstan adjacent to the coastal sites where the epizootics of 1997 and 2000 originated. Increased focus is also warranted on the development of robust in vitro assays and additional in vivo assays to better understand the interspecies transmission of CDV, especially with respect to the use of heterologous CD150s and Nectin 4s and the identification of additional viral and host factors restricting cross-species infections. Given that eradication of CDV is not currently a viable prospect, obtaining a better understanding of CDV cross-species infections will better inform assessments of the risk of zoonotic infections in the event of a successful measles eradication campaign.

## Figures and Tables

**Table 1:** List of canine distemper virus (CDV)-infected samples used for next-generation sequence analysis.

Clade	Strain Variant	Stranding Year	Location	Host	Sample Material	Coverage	GenBank Accession No.
Caspian	PC00-46	2000	Kazakhstan	Caspian seal	Kidney	393x	MN267064
Caspian	PC00-20	2000	Kazakhstan	Caspian seal	Kidney	547x	--
Caspian	PC00-20	2000	Kazakhstan	Caspian seal	Lung	9149x	MN267065
Caspian	PC00-21	2000	Azerbaijan	Caspian seal	Lung	33184x	MN267066
Caspian	PC00-21	2000	Azerbaijan	Caspian seal	Kidney	0.3x	--
Arctic	PS88-428	1988	Russia	Baikal seal	Lung	2572x	MN267063
Arctic	PS88-428	1988	Russia	Baikal seal	Spleen	247x	--
Europe-1	S466	2015	Germany	Raccoon	Lung	19505x	MN267062
Europe-1	S460	2015	Germany	Raccoon	Lung	9922x	MN267060
Europe-1	S272	2016	Germany	Fox	Tonsils	157x	MN267061

**Table 2:** Unique amino acid changes in the genome strains generated from this study compared to 94 CDV full-length sequences.

Protein	PC00-46/20/21 (Caspian seal)		PS88-428 (Baikal seal)	S460/S466/S272 (Europe-1)
**Nucleocapsid**	N**65**S		A**140**V	D**151**N
	D**135**N			P/L**432**S
	S**439**F			T/A/S**434**V
	G**458**E			R**438**S
	Y**459**H			L/F/P/V**456**S
	Y/F/H**471**P			V**463**A
				E/G**467**K
**Phosphoprotein**	I**29**V	P**223**S	H/Y**75**D	E**54**D
	N/D**46**S	S**238**L	D**206**E	Q**102**H
	T/I/A**48**G	S**254**P	G/S**418**N	T**209**I
	M/V/K**51**I	V**272**A	D**443**N	
	K/R/E**68**T	A**458**P		
	N/S**138**D			
**Matrix**	No unique changes		No unique changes	S**202**L
**Fusion**	K/N/E/R**3**G	R/K**73**G	**V/A/T**32**I**	I/T/S/V**30**A
	E/K**7**T	S/L**74**P	R/M/G**65**I	I/T**33**V/A
	Q/R**22**H	V/I/F**87**T	S/T**91**P	V/I/D**57**T
	**V/A/T32I**	V/I**94**A	I/T/V**110**F	A/G**85**C
	Q/H/R/L**43**K	I/L**124**F		I**515**M
	H/R/Y**47**D	P**161**S		N**517**S
	T/I/A**52**K	K**211**R		P**613**L
	C/R**67**H	P/L**212**S		
	Q**69**H	D/A**644**N		
	A/T**70**I			
**Hemagglutinin**	P**35**S	T**360**I	A**191**V	S**311**P
	K/R**161**N	E**379**K	R/K**197**E	D/N**313**S
	L**175**I	S**394**A	D**571**G	V**334**M
	G/R**177**E	I/T/V**417**A		M**362**V
	G/D/A**178**S	E/D**441**K		M/V**389**I
	V/I/S**198**G	S/P**447**F		Q**516**R
	P/S**200**L	G**488**R		T**590**I
	I**210**V	S**497**P		R/C/H/S**597**Y
	D/N**237**Y	R/I**519**K		
	T/S**245**A	E/D**560**N		
	T**268**I	D/N**584**T		
	Y/S**305**H	K/N**606**S		
	N/S/D/R**309**K			
**Large**	A**32**S	K**1229**R	N**319**T	V**6**I
	N/S**37**R	N/H**1391**I	R**699**K	C/R**65**Y
	T**43**A	S/I/V**1392**T	V/I**975**M	V**85**M
	R**47**C	V**1445**I		V/A**256**G
	I**66**V	Q/R**1701**S		D**318**V
	S/A/P**252**T	S/P**1702**L		Y**400**F
	M/I**385**L	S**1995**T		T/A/S/I**622**G
	R/C/Y/S/W**611**H	S**2023**G		D**717**N
	K/R**615**Q	N**2141**H		I**917**V
	L**1179**I			F**1718**L
				N/T**1776**K
				I**2024**M
				L**2175**V
**Total**	80 changes		15 changes	39 changes

## Supplementary Materials

The following are available online at https://www.mdpi.com/1999-4915/11/10/894/s1, Table S1: CDV lineages Hemagglutinin–SLAMF1 interaction sites, Table S2: CDV lineages Hemagglutinin–Nectin-4 interaction sites, Table S3: Genomic differences between the three recovered Caspian strains using as reference strain PC00-46, Figure S1: Maximum-likelihood phylogenetic tree of CDV full-length genomes, Figure S2: Bayesian phylogenetic tree of CDV full-length genomes, Figure S3: Bayesian phylogenetic tree of CDV-full length genomes without modified live vaccines, Table S4: Major recombination events identified by all detection methods in RDP4, Figure S4: Temporal signal analysis of CDV full-length genomes, Figure S5: Maximum-likelihood tree of complete CDV-H gene sequences.

## References

[1] Martinez-Gutierrez M., Ruiz-Saenz J. (2016). Diversity of susceptible hosts in canine distemper virus infection: A systematic review and data synthesis. BMC Veter Res..

[2] Sun Z., Li A., Ye H., Shi Y., Hu Z., Zeng L. (2010). Natural infection with canine distemper virus in hand-feeding Rhesus monkeys in China. Veter Microbiol..

[3] Qiu W., Zheng Y., Zhang S., Fan Q., Liu H., Zhang F., Wang W., Liao G., Hu R. (2011). Canine Distemper Outbreak in Rhesus Monkeys, China. Emerg. Infect. Dis..

[4] Sakai K., Nagata N., Ami Y., Seki F., Suzaki Y., Iwata-Yoshikawa N., Suzuki T., Fukushi S., Mizutani T., Yoshikawa T. (2013). Lethal canine distemper virus outbreak in cynomolgus monkeys in Japan in 2008. J. Virol..

[5] Appel M.J., Reggiardo C., Summers B.A., Pearce-Kelling S., Maré C.J., Noon T.H., Reed R.E., Shively J.N., Orvell C. (1991). Canine distemper virus infection and encephalitis in javelinas (collared peccaries). Arch. Virol..

[6] Origgi F.C., Sattler U., Pilo P., Waldvogel A.S. (2013). Fatal Combined Infection With Canine Distemper Virus and Orthopoxvirus in a Group of Asian Marmots (Marmota caudata). Veter Pathol..

[7] Lunardi M., Darold G.M., Amude A.M., Headley S.A., Sonne L., Yamauchi K.C.I., Boabaid F.M., Alfieri A.F., Alfieri A.A. (2018). Canine distemper virus active infection in order Pilosa, family Myrmecophagidae, species Tamandua tetradactyla. Veter Microbiol..

[8] Ke G.-M., Ho C.-H., Chiang M.-J., Sanno-Duanda B., Chung C.-S., Lin M.-Y., Shi Y.-Y., Yang M.-H., Tyan Y.-C., Liao P.-C. (2015). Phylodynamic analysis of the canine distemper virus hemagglutinin gene. BMC Veter Res..

[9] Riley M.C., Wilkes R.P. (2015). Sequencing of emerging canine distemper virus strain reveals new distinct genetic lineage in the United States associated with disease in wildlife and domestic canine populations. Virol. J..

[10] Keller S.M., Gabriel M., Terio K.A., Dubovi E.J., VanWormer E., Sweitzer R., Barret R., Thompson C., Purcell K., Munson L. (2012). Canine distemper in an isolated population of fishers (Martes pennanti) from California. J. Wildl. Dis..

[11] Anis E., Newell T.K., Dyer N., Wilkes R.P. (2018). Phylogenetic analysis of the wild-type strains of canine distemper virus circulating in the United States. Virol. J..

[12] Panzera Y., Calderón M.G., Sarute N., Guasco S., Cardeillac A., Bonilla B., Hernández M., Francia L., Bedó G., La Torre J. (2012). Evidence of two co-circulating genetic lineages of canine distemper virus in South America. Virus Res..

[13] Espinal M.A., Díaz F.J., Ruiz-Saenz J. (2014). Phylogenetic evidence of a new canine distemper virus lineage among domestic dogs in Colombia, South America. Veter Microbiol..

[14] Piewbang C., Radtanakatikanon A., Puenpa J., Poovorawan Y., Techangamsuwan S. (2019). Genetic and evolutionary analysis of a new Asia-4 lineage and naturally recombinant canine distemper virus strains from Thailand. Sci. Rep..

[15] Bhatt M., Rajak K.K., Chakravarti S., Yadav A.K., Kumar A., Gupta V., Chander V., Mathesh K., Chandramohan S., Sharma A.K. (2019). Phylogenetic analysis of haemagglutinin gene deciphering a new genetically distinct lineage of canine distemper virus circulating among domestic dogs in India. Transbound. Emerg. Dis..

[16] Zhao J.-J., Yan X.-J., Chai X.-L., Martella V., Luo G.-L., Zhang H.-L., Gao H., Liu Y.-X., Bai X., Zhang L. (2010). Phylogenetic analysis of the haemagglutinin gene of canine distemper virus strains detected from breeding foxes, raccoon dogs and minks in China. Veter Microbiol..

[17] Iwatsuki K., Miyashita N., Yoshida E., Gemma T., Shin Y.S., Mori T., Hirayama N., Kai C., Mikami T. (1997). Molecular and phylogenetic analyses of the haemagglutinin (H) proteins of field isolates of canine distemper virus from naturally infected dogs. J. Gen. Virol..

[18] Pardo I.D.R., Johnson G.C., Kleiboeker S.B. (2005). Phylogenetic characterization of canine distemper viruses detected in naturally infected dogs in North America. J. Clin. Microbiol..

[19] Sarute N., Calderón M.G., Pérez R., La Torre J., Hernández M., Francia L., Panzera Y. (2013). The fusion protein signal-peptide-coding region of canine distemper virus: A useful tool for phylogenetic reconstruction and lineage identification. PLoS ONE.

[20] Ludlow M., Rennick L.J., Nambulli S., De Swart R.L., Paul Duprex W. (2014). Using the ferret model to study morbillivirus entry, spread, transmission and cross-species infection. Curr. Opin. Virol..

[21] Lemon K., de Vries R.D., Mesman A.W., McQuaid S., van Amerongen G., Yüksel S., Ludlow M., Rennick L.J., Kuiken T., Rima B.K. (2011). Early target cells of measles virus after aerosol infection of non-human primates. PLoS Pathog..

[22] Von Messling V., Milosevic D., Cattaneo R. (2004). Tropism illuminated: Lymphocyte-based pathways blazed by lethal morbillivirus through the host immune system. Proc. Natl. Acad. Sci. USA.

[23] Tatsuo H., Ono N., Yanagi Y. (2001). Morbilliviruses use signaling lymphocyte activation molecules (CD150) as cellular receptors. J. Virol..

[24] Von Messling V., Springfeld C., Devaux P., Cattaneo R. (2003). A ferret model of canine distemper virus virulence and immunosuppression. J. Virol..

[25] Noyce R.S., Delpeut S., Richardson C.D. (2013). Dog nectin-4 is an epithelial cell receptor for canine distemper virus that facilitates virus entry and syncytia formation. Virology.

[26] Sawatsky B., Wong X.-X., Hinkelmann S., Cattaneo R., von Messling V. (2012). Canine distemper virus epithelial cell infection is required for clinical disease but not for immunosuppression. J. Virol..

[27] Summers B.A., Greisen H.A., Appel M.J. (1984). Canine distemper encephalomyelitis: Variation with virus strain. J. Comp. Pathol..

[28] Axthelm M.K., Krakowka S. (1998). Experimental old dog encephalitis (ODE) in a gnotobiotic dog. Veter Pathol..

[29] Grachev M.A., Kumarev V.P., Mamaev L.V., Zoryn V.L., Baranova L., Denikina N.N., Belikov S.I., Petrov E.A., Kolesnik V.S., Kolesnik R.S. (1989). Distemper virus in Baikal seals. Nature.

[30] Forsyth M.A., Kennedy S., Wilson S., Eybatov T., Barrett T. (1998). Canine distemper virus in a Caspian seal. Veter Rec..

[31] Kennedy S., Kuiken T., Jepson P., Deaville R., Forsyth M., Barrett T., van de Bildt M., Osterhaus A., Eybatov T., Duck C. (2000). Mass Die-Off of Caspian Seals Caused by Canine Distemper Virus. Emerg. Infect. Dis..

[32] Goodman S.J., Dmitrieva L. Pusa Caspica. http://www.iucnredlist.org/details/41669/0.

[33] Mamaev L.V., Denikina N.N., Belikov S.I., Volchkov V.E., Visser I.K., Fleming M., Kai C., Harder T.C., Liess B., Osterhaus A.D. (1995). Characterisation of morbilliviruses isolated from Lake Baikal seals (Phoca sibirica). Veter Microbiol..

[34] Stanton J.B., Brown C.C., Poet S., Lipscomb T.P., Saliki J., Frasca S. (2004). Retrospective differentiation of canine distemper virus and phocine distemper virus in phocids. J. Wildl. Dis..

[35] Jo W.K., Kruppa J., Habierski A., van de Bildt M., Mazzariol S., Di Guardo G., Siebert U., Kuiken T., Jung K., Osterhaus A. (2018). Evolutionary evidence for multi-host transmission of cetacean morbillivirus. Emerg. Microbes Infect..

[36] Katoh K., Rozewicki J., Yamada K.D. (2017). MAFFT online service: Multiple sequence alignment, interactive sequence choice and visualization. Brief. Bioinform..

[37] Kumar S., Stecher G., Tamura K. (2016). MEGA7: Molecular Evolutionary Genetics Analysis Version 7.0 for Bigger Datasets. Mol. Biol. Evol..

[38] Rambaut A., Lam T.T., Max Carvalho L., Pybus O.G. (2016). Exploring the temporal structure of heterochronous sequences using TempEst (formerly Path-O-Gen). Virus Evol..

[39] Martin D.P., Murrell B., Golden M., Khoosal A., Muhire B. (2015). RDP4: Detection and analysis of recombination patterns in virus genomes. Virus Evol..

[40] Drummond A., Bouckaert R. (2015). Bayesian Evolutionary Analysis with BEAST.

[41] Stamatakis A. (2014). RAxML version 8: A tool for phylogenetic analysis and post-analysis of large phylogenies. Bioinformatics.

[42] Hashiguchi T., Ose T., Kubota M., Maita N., Kamishikiryo J., Maenaka K., Yanagi Y. (2011). Structure of the measles virus hemagglutinin bound to its cellular receptor SLAM. Nat. Struct. Mol. Biol..

[43] Zhang X., Lu G., Qi J., Li Y., He Y., Xu X., Shi J., Zhang C.W.-H., Yan J., Gao G.F. (2013). Structure of measles virus hemagglutinin bound to its epithelial receptor nectin-4. Nat. Struct. Mol. Biol..

[44] Rieux A., Balloux F. (2016). Inferences from tip-calibrated phylogenies: A review and a practical guide. Mol. Ecol..

[45] Gámiz C., Martella V., Ulloa R., Fajardo R., Quijano-Hernandéz I., Martínez S., Martella V. (2011). Identification of a new genotype of canine distemper virus circulating in America. Veter Res. Commun..

[46] Dudas G., Bedford T. (2019). The ability of single genes vs full genomes to resolve time and space in outbreak analysis. bioRxiv.

[47] Yuan C., Liu W., Wang Y., Hou J., Zhang L., Wang G. (2017). Homologous recombination is a force in the evolution of canine distemper virus. PLoS ONE.

[48] Da Fontoura Budaszewski R., Streck A.F., Nunes Weber M., Maboni Siqueira F., Muniz Guedes R.L., Wageck Canal C. (2016). Influence of vaccine strains on the evolution of canine distemper virus. Infect. Genet. Evol..

[49] Blancou J. (2004). Dog distemper: Imported into Europe from South America?. Hist. Med. Veter.

[50] Kirk H. (1922). Canine Distemper: Its Complications, Sequelae, and Treatment.

[51] Green R.G. (1925). Distemper in the silver fox (Culpes vulpes). Exp. Biol. Med..

[52] Puntoni V. (1923). Saggio di vaccinazione anticimurrosa preventiva eseguita per mezzo del virus specifico. Ann. Igiene.

[53] Bresalier M., Worboys M. (2014). ‘Saving the lives of our dogs’: The development of canine distemper vaccine in interwar Britain. Br. J. Hist. Sci..

[54] Ek-Kommonen C., Sihvonen L., Pekkanen K., Rikula U., Nuotio L. (1997). Outbreak of canine distemper in vaccinated dogs in Finland. Veter Rec..

[55] Martella V., Cirone F., Elia G., Lorusso E., Decaro N., Campolo M., Desario C., Lucente M.S., Bellacicco A.L., Blixenkrone-Møller M. (2006). Heterogeneity within the hemagglutinin genes of canine distemper virus (CDV) strains detected in Italy. Veter Microbiol..

[56] Feng N., Yu Y., Wang T., Wilker P., Wang J., Li Y., Sun Z., Gao Y., Xia X. (2016). Fatal canine distemper virus infection of giant pandas in China. Sci. Rep..

[57] Sulikhan N.S., Gilbert M., Blidchenko E.Y., Naidenko S.V., Ivanchuk G.V., Gorpenchenko T.Y., Alshinetskiy M.V., Shevtsova E.I., Goodrich J.M., Lewis J.C.M. (2017). Canine Distemper Virus in a Wild Far Eastern Leopard Panthera pardus orientalis. J. Wildl. Dis..

[58] Bolt G., Jensen T.D., Gottschalck E., Arctander P., Appel M.J.G., Buckland R., Blixenkrone-Møller M. (1997). Genetic diversity of the attachment (H) protein gene of current field isolates of canine distemper virus. J. Gen. Virol..

[59] Kapil S., Allison R.W., Johnston L., Murray B.L., Holland S., Meinkoth J., Johnson B. (2008). Canine Distemper Virus Strains Circulating among North American Dogs. Clin. Vaccine Immunol..

[60] Namroodi S., Rostami A., Majidzadeh-Ardebili K., Ghalyanchi Langroudi A., Morovvati A. (2015). Detection of Arctic and European cluster of canine distemper virus in north and center of Iran. Vet. Res. Forum.

[61] Di Sabatino D., Di Francesco G., Zaccaria G., Malatesta D., Brugnola L., Marcacci M., Portanti O., De Massis F., Savini G., Teodori L. (2016). Lethal distemper in badgers (Meles meles) following epidemic in dogs and wolves. Infect. Genet. Evol..

[62] Butina T.V., Denikina N.N., Belikov S.I. (2010). Canine distemper virus diversity in Lake Baikal seal (Phoca sibirica) population. Veter Microbiol..

[63] Panzera Y., Sarute N., Iraola G., Hernández M., Pérez R. (2015). Molecular phylogeography of canine distemper virus: Geographic origin and global spreading. Mol. Phylogenet. Evol..

[64] Pomeroy L.W., Bjørnstad O.N., Holmes E.C. (2008). The Evolutionary and Epidemiological Dynamics of the Paramyxoviridae. J. Mol. Evol..

[65] Duque-Valencia J., Sarute N., Olarte-Castillo X.A., Ruíz-Sáenz J. (2019). Evolution and Interspecies Transmission of Canine Distemper Virus—An Outlook of the Diverse Evolutionary Landscapes of a Multi-Host Virus. Viruses.

