# Supplementary material for: The Canine Morbillivirus Strain Associated with An Epizootic in Caspian Seals Provides New Insights into the Evolutionary History of this Virus
Source: Viruses. 2019 Sep 25;11(10):894. doi: 10.3390/v11100894 (PMC6832514; doi:10.3390/v11100894)
Supplement: Supplementary file 1 [file viruses-11-00894-s001.zip › TableS1_SLAM binding sites.pdf]

SLAM

Lineage	Strain	Site I		Site II						Site III					Site IV					
		501	503	526	527	528	529	548	550	191	192	193	194	195	520	537	539	548	479	541
PDV	KC802221_PDV_WaddenSea_seal_NL_1988	D	D	D	V	S	R	T	P	A	T	T	S	M	Y	Y	Y	T	L	P
**Caspian**	**PC0046_seal_KZ_2000**	**D**	**D**	**D**	**I**	**S**	**R**	**T**	**P**	**A**	**T**	**T**	**S**	**V**	**Y**	**Y**	**Y**	**T**	**L**	**P**
	**PC0021_seal_AZ_2000**	**D**	**D**	**D**	**I**	**S**	**R**	**T**	**P**	**A**	**T**	**T**	**S**	**V**	**Y**	**Y**	**Y**	**T**	**L**	**P**
	**PC0020_seal_KZ_2000**	**D**	**D**	**D**	**I**	**S**	**R**	**T**	**P**	**A**	**T**	**T**	**S**	**V**	**Y**	**Y**	**Y**	**T**	**L**	**P**
America-1	JN896987_SnyderHill_1950	D	D	D	I	S	R	T	P	A	T	T	S	V	Y	Y	Y	T	L	P
	AF378705_Onderstepoort_1939	D	D	D	I	S	R	T	P	A	T	T	S	V	Y	Y	Y	T	L	P
Europe-1	**S460_raccoon_DE_2015**	**D**	**D**	**D**	**I**	**S**	**R**	**T**	**P**	**A**	**T**	**T**	**S**	**V**	**Y**	**Y**	**Y**	**T**	**L**	**P**
	**S466_raccoon_DE_2015**	**D**	**D**	**D**	**I**	**S**	**R**	**T**	**P**	**A**	**T**	**T**	**S**	**V**	**Y**	**Y**	**Y**	**T**	**L**	**P**
	**S272_fox_DE_2016**	**D**	**D**	**D**	**I**	**S**	**R**	**T**	**P**	**A**	**T**	**T**	**S**	**V**	**Y**	**Y**	**Y**	**T**	**L**	**P**
	AY386315_5804_dogV_DE_1989	D	D	D	I	S	R	T	P	A	T	T	S	V	Y	Y	Y	T	L	P
America-3	KF640687_R252_dogV_US_1974	D	D	D	I	S	R	T	P	A	T	T	S	V	Y	Y	Y	T	L	P
America-2	KU666057_RhodeIsland_raccoon_US_2012	D	D	D	I	S	R	T	P	A	T	T	S	V	Y	Y	Y	T	L	P
Africa-1	KU578256_SNP_lion_TZ_1994	D	D	D	V	S	R	T	P	A	T	T	S	V	Y	Y	Y	T	L	P
Asia-4	MH496773_2_dog_TH_2014	D	D	D	I	S	R	T	P	A	T	T	S	V	Y	Y	Y	T	L	P
Arctic	**Baikal_seal_RU_1988**	**D**	**D**	**D**	**I**	**S**	**R**	**T**	**P**	V	**T**	**T**	**S**	**V**	**Y**	**Y**	**Y**	**T**	**L**	**P**
	KX024708_11956_badger_IT_2015	D	D	D	I	S	R	T	P	A	T	T	S	V	Y	Y	Y	T	L	P
Asia-2	AB475097_M25CR_dog_JP_2009	D	D	D	I	S	R	T	P	A	T	T	S	V	Y	Y	Y	T	L	P
Asia-1	KC427278_Hebei_mink_CN_2008	D	D	D	I	S	R	T	P	A	T	T	S	V	Y	Y	Y	T	L	P
	MK431532_NTU_dog_TW_2005	D	D	D	I	S	R	T	P	A	T	T	S	V	Y	Y	Y	T	L	P
	MH496776_5_dog_TH_2014	D	D	D	I	S	R	T	P	A	T	T	S	V	Y	Y	Y	T	L	P
	MF041963_CDV06_wolf_ET_2016	D	N	D	I	S	R	T	P	A	T	T	S	V	Y	Y	Y	T	L	P
	KP765764_LN1_fox_CN_2010	D	D	D	I	S	R	T	P	A	T	T	S	V	Y	Y	Y	T	L	P
	KP769803_Shaanxi_panda_CN_2015	D	D	D	I	S	R	T	P	A	T	T	S	V	Y	Y	Y	T	L	P
	KP738610_SD11_raccoondog_CN_2014	D	D	D	I	S	R	T	P	A	T	T	S	V	Y	Y	Y	T	L	P
	HM852904_MKYKM08_macaque_CN_2008	D	D	D	I	S	R	T	P	A	T	T	S	V	Y	Y	Y	T	L	P
	LC342298_RC13_raccoon_JP_2016	D	D	D	I	S	R	T	P	A	T	T	S	V	Y	Y	Y	T	L	P
Africa-2	KY971532_WT02_hyena_SA_2017	D	D	D	I	S	R	T	P	A	T	T	S	I	Y	Y	Y	T	L	P

Sites are based on MV-H interaction with marmoset SLAM (Hashiguchi et al 2011)
