# Supplementary material for: The Canine Morbillivirus Strain Associated with An Epizootic in Caspian Seals Provides New Insights into the Evolutionary History of this Virus
Source: Viruses. 2019 Sep 25;11(10):894. doi: 10.3390/v11100894 (PMC6832514; doi:10.3390/v11100894)
Supplement: Supplementary file 1 [file viruses-11-00894-s001.zip › TableS2_PVRL4 binding sites.pdf]

Nectin-4

Lineage	Strain	Site I												Site II				Site III					
		520	522	537	539	454	455	458	459	460	461	478	479	494	495	501	496	383	384	385	387	495	496
PDV	KC802221_PDV_WaddenSea_seal_NL_1988	Y	V	Y	Y	P	K	T	I	L	G	I	L	I	Q	D	T	K	R	R	Y	Q	T
**Caspian**	**PC0046_seal_KZ_2000**	Y	I	Y	Y	P	K	T	I	L	G	V	L	I	Q	D	T	Q	R	K	Y	Q	T
	**PC0021_seal_AZ_2000**	Y	I	Y	Y	P	K	T	I	L	G	V	L	I	Q	D	T	Q	R	K	Y	Q	T
	**PC0020_seal_KZ_2000**	Y	I	Y	Y	P	K	T	I	L	G	V	L	I	Q	D	T	Q	R	K	Y	Q	T
America-1	JN896987_SnyderHill_1950	Y	I	Y	Y	P	K	T	I	L	G	V	L	I	Q	D	T	Q	R	K	Y	Q	T
	AF378705_Onderstepoort_1939	Y	I	Y	Y	P	K	T	I	V	G	V	L	I	Q	D	T	Q	R	K	Y	Q	T
Europe-1	**S460_raccoon_DE_2015**	Y	I	Y	Y	P	K	T	V	L	G	V	L	I	Q	D	T	Q	R	K	Y	Q	T
	**S466_raccoon_DE_2015**	Y	I	Y	Y	P	K	T	V	L	G	V	L	I	Q	D	T	Q	R	K	Y	Q	T
	**S272_fox_DE_2016**	Y	I	Y	Y	P	K	T	V	L	G	V	L	I	Q	D	T	Q	R	K	Y	Q	T
	AY386315_5804_dogV_DE_1989	Y	I	Y	Y	P	R	T	V	L	G	V	L	I	Q	D	T	Q	R	K	Y	Q	T
America-3	KF640687_R252_dogV_US_1974	Y	I	Y	Y	P	K	T	V	L	G	V	L	I	Q	D	T	Q	R	K	Y	Q	T
America-2	KU666057_RhodeIsland_raccoon_US_2012	Y	I	Y	Y	P	M	T	V	L	G	V	L	I	Q	D	T	Q	R	K	Y	Q	T
Africa-1	KU578256_SNP_lion_TZ_1994	Y	I	Y	Y	P	K	T	V	L	G	V	L	I	Q	D	T	Q	R	K	Y	Q	T
Asia-4	MH496773_2_dog_TH_2014	Y	I	Y	Y	P	K	T	V	L	G	V	L	I	Q	D	T	Q	R	K	Y	Q	T
Arctic	**Baikal_seal_RU_1988**	Y	I	Y	Y	P	K	T	I	L	G	V	L	I	Q	D	T	Q	R	K	Y	Q	T
	KX024708_11956_badger_IT_2015	Y	I	Y	Y	P	K	T	I	L	G	V	L	I	Q	D	T	Q	R	K	Y	Q	T
Asia-2	AB475097_M25CR_dog_JP_2009	Y	V	Y	Y	P	K	T	I	L	G	V	L	I	Q	D	T	Q	R	K	Y	Q	T
Asia-1	KC427278_Hebei_mink_CN_2008	Y	I	Y	Y	P	K	T	V	L	G	V	L	I	Q	D	T	H	R	K	Y	Q	T
	MK431532_NTU_dog_TW_2005	Y	I	Y	Y	P	K	T	V	L	G	V	L	I	Q	D	T	Q	R	K	Y	Q	T
	MH496776_5_dog_TH_2014	Y	I	Y	Y	P	K	T	V	L	G	V	L	I	Q	D	T	H	R	K	Y	Q	T
	MF041963_CDV06_wolf_ET_2016	Y	I	Y	Y	P	K	T	V	L	G	V	L	I	Q	D	T	H	R	K	Y	Q	T
	KP765764_LN1_fox_CN_2010	Y	I	Y	Y	P	K	T	V	L	G	V	L	I	Q	D	T	Q	R	K	Y	Q	T
	KP769803_Shaanxi_panda_CN_2015	Y	I	Y	Y	P	K	T	V	L	G	V	L	I	Q	D	T	Q	R	K	Y	Q	T
	KP738610_SD11_raccoondog_CN_2014	Y	I	Y	Y	P	K	T	V	L	G	V	L	I	Q	D	T	Q	R	K	Y	Q	T
	HM852904_MKYKM08_macaque_CN_2008	Y	I	Y	Y	P	K	T	V	L	G	V	L	I	Q	D	T	Q	R	K	Y	Q	T
	LC342298_RC13_raccoon_JP_2016	Y	I	Y	Y	P	K	T	V	L	G	V	L	I	Q	D	T	Q	R	K	Y	Q	T
Africa-2	KY971532_WT02_hyena_SA_2017	Y	V	Y	Y	P	K	T	I	L	G	V	L	I	Q	D	T	Q	R	K	Y	Q	T

Sites are based on MV-H interaction with human nectin-4 (Zhang et al 2013)
