# Supplementary material for: The Canine Morbillivirus Strain Associated with An Epizootic in Caspian Seals Provides New Insights into the Evolutionary History of this Virus
Source: Viruses. 2019 Sep 25;11(10):894. doi: 10.3390/v11100894 (PMC6832514; doi:10.3390/v11100894)
Supplement: Supplementary file 1 [file viruses-11-00894-s001.zip › TableS3_PC00-changes_new.pdf]

Table S3. Genomic differences between the three recovered Caspian strain variants

Region	Nt pos	AA pos	Strain variant					
			PC00-46		PC00-20		PC00-21	
			Nt	AA	Nt	AA	Nt	AA
N	146		G		A		A	
	1250		C		T		T	
	1496		C		T		C	
P/V/C	2069	90	C	P	T	L	T	L
M	3458		A		G		G	
F	5311	126	T	M	C	T	C	T
	5390		C		T		C	
	6987		T		G		G	
H	7320		T		C		C	
	8308	410	A	I	G	M	A	I
L	9752		C		T		T	
	10148		C		T		T	
	10523		T		T		A	
	11276		T		T		C	
	11423		G		A		A	
	12266		A		G		G	
	13427		C		T		T	
	13808		T		C		C	
	15035		A		G		G	

Blank spaces indicate no change or not applicable
