# Supplementary material for: The Canine Morbillivirus Strain Associated with An Epizootic in Caspian Seals Provides New Insights into the Evolutionary History of this Virus
Source: Viruses. 2019 Sep 25;11(10):894. doi: 10.3390/v11100894 (PMC6832514; doi:10.3390/v11100894)
Supplement: Supplementary file 1 [file viruses-11-00894-s001.zip › TableS4_Recombination_new.pdf]

**Table S4.** Major recombination events identified by all detection methods in RDP4

| Recombinant (Accession No.) | Major parent (Accession No.) | Minor parent (Accession No.) | Breakpoint positions | Detection methods (*p*-value) | | | | | | |
|---|---|---|---|---|---|---|---|---|---|---|
| | | | | RDP | GENECONV | BootScan | MaxChi | Chimaera | SiScan | 3Seq |
| HLJ1 (JX681125) | TH-8 (MH496779) 98.9% similarity | Phoca/Caspian/2007 (HM046486) 99.9% similarity | 12926 – 14264 | 1.58E-72 | 3.39E-70 | 6.38E-73 | 1.75E-16 | 3.12E-16 | 1.66E-19 | 4.32E-11 |
| 2645 (AY445077) 2654 (AY466011) 2645 (AY542312) | 2689 (AY649446) 98% similarity | Snyder Hill (JN896987) 98.9% similarity | 7131 – 9032 | 2.85E-48 | 7.68E-47 | 4.65E-50 | 2.89E-19 | 1.25E-05 | 8.31E-27 | 2.88E-11 |
| ZC (KJ994343) | PS (JN896331) 98% similarity | Onderstepoort (AF305419) 100% similarity | 11586 – 12022 | 1.44E-32 | 3.70E-31 | 9.45E-33 | 3.10E-05 | 2.46E-05 | 8.96E-07 | 4.32E-11 |
| 007Lm (AB474397) | 011C (AB476401) 99.5% similarity | 2689 (AY649446) 99.8% similarity | 7054 – 7555 | 2.98E-27 | 1.51E-25 | 2.09E-23 | 6.68E-08 | 1.80E-07 | 2.58E-07 | 2.88E-11 |
| 007Lm (AB474397) | 011C (AB476401) 99.7% similarity | 2689 (AY649446) 99.9% similarity | 9794 – 10563 | 1.68E-27 | 2.21E-26 | 1.59E-27 | 1.18E-06 | 1.16E-06 | 5.22E-07 | 2.88E-11 |
| 2689 (AY649446) | 2601 (AY443350) 99% similarity | 164071 (EU716337) 99.8% similarity | 4940 – 6962 | 4.32E-19 | 6.93E-18 | 1.56E-20 | 2.20E-11 | 1.54E-12 | 4.91E-13 | 2.28E-16 |
| ZC (KJ994343) | Unknown strain (171391513_KJ123771) | 164071 (EU716337) 99.4% similarity | 12925 – 14137 | 1.06E-10 | 3.56E-10 | 5.50E-08 | 1.74E-08 | 2.95E-08 | 5.27E-06 | 2.02E-07 |
| 171391513 (KJ123771) | Unknown strain (A7517_AF164967) | 164071 (EU716337) 100% similarity | 2008 – 5718 | 6.70E-09 | 5.90E-07 | 6.74E-09 | 9.54E-06 | 3.78E-05 | 3.37E-05 | 2.43E-06 |
| 2654 (AY466011) | 164071 (EU716337) 97.8% similarity | CDV3 (EU726268) 99.5% similarity | 2134 – 2556 | 1.26E-07 | 3.95E-07 | 1.35E-07 | 1.63E-03 | 4.87E-04 | 7.20E-03 | 1.60E-04 |
| CDV4_TH (MH496775) | CDV8_TH (MH496779) 99% similarity | 171391513 (KJ123771) 100% similarity | 8250 – 8987 | 7.05E-09 | 1.71E-10 | 5.96E-12 | 0.0446697 | NS | 0.0001756 | 2.14E-06 |
