# Supplementary figures and images for: The Canine Morbillivirus Strain Associated with An Epizootic in Caspian Seals Provides New Insights into the Evolutionary History of this Virus
Source: Viruses. 2019 Sep 25;11(10):894. doi: 10.3390/v11100894 (PMC6832514; doi:10.3390/v11100894)

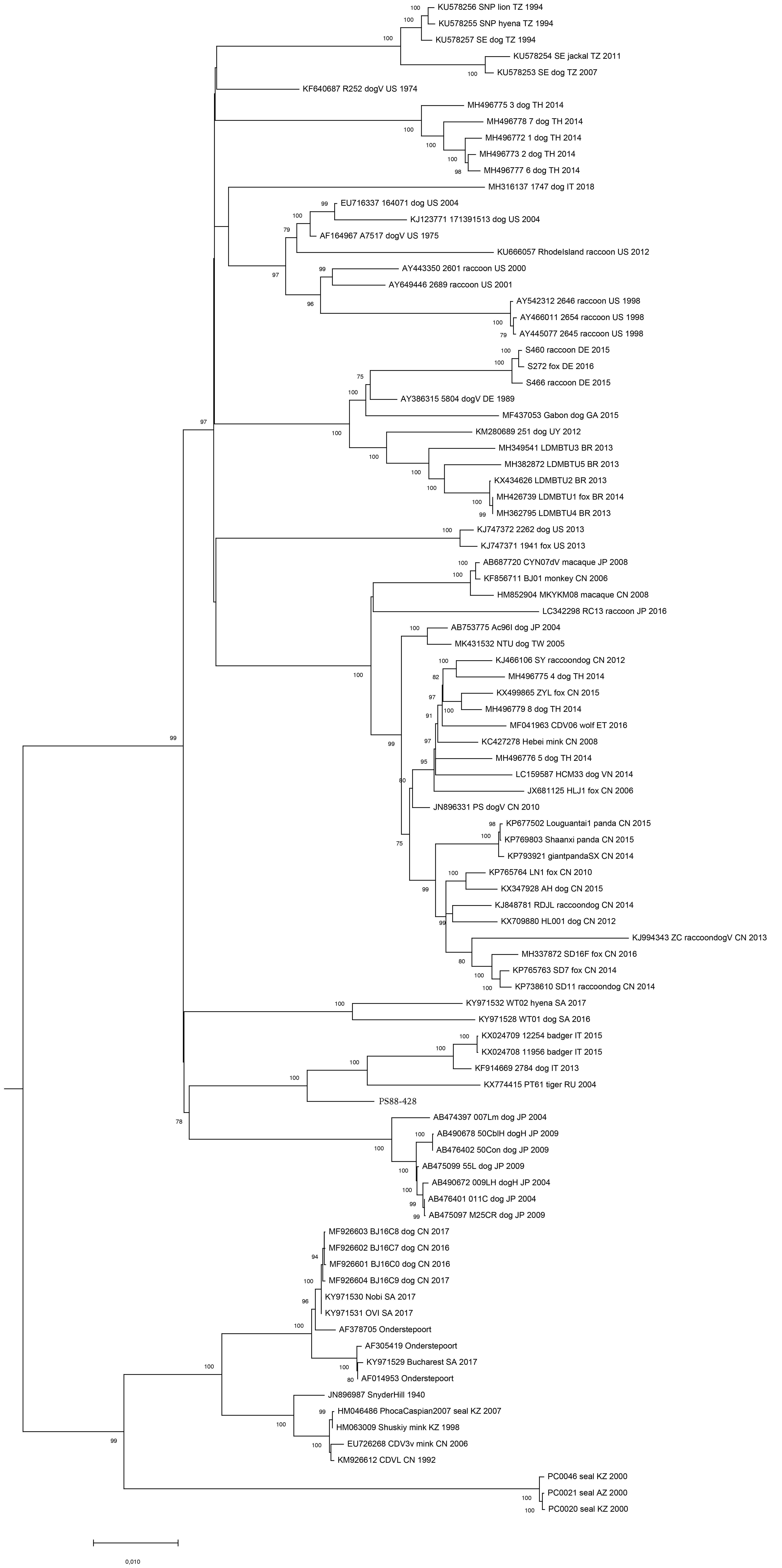

Supplement: Supplementary file 1 [file viruses-11-00894-s001.zip › Fig-S1.jpg]

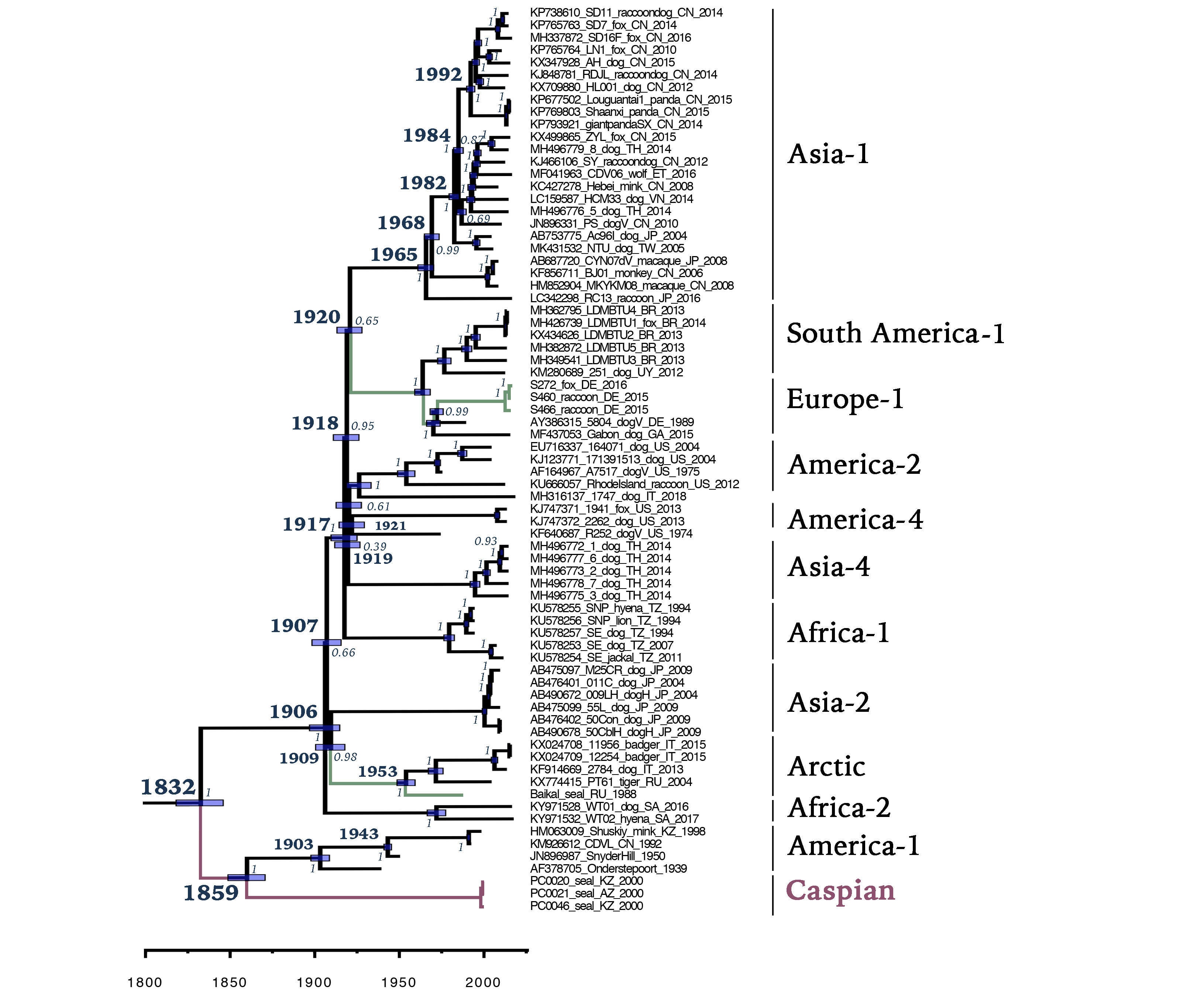

Supplement: Supplementary file 1 [file viruses-11-00894-s001.zip › Fig-S2.jpg]

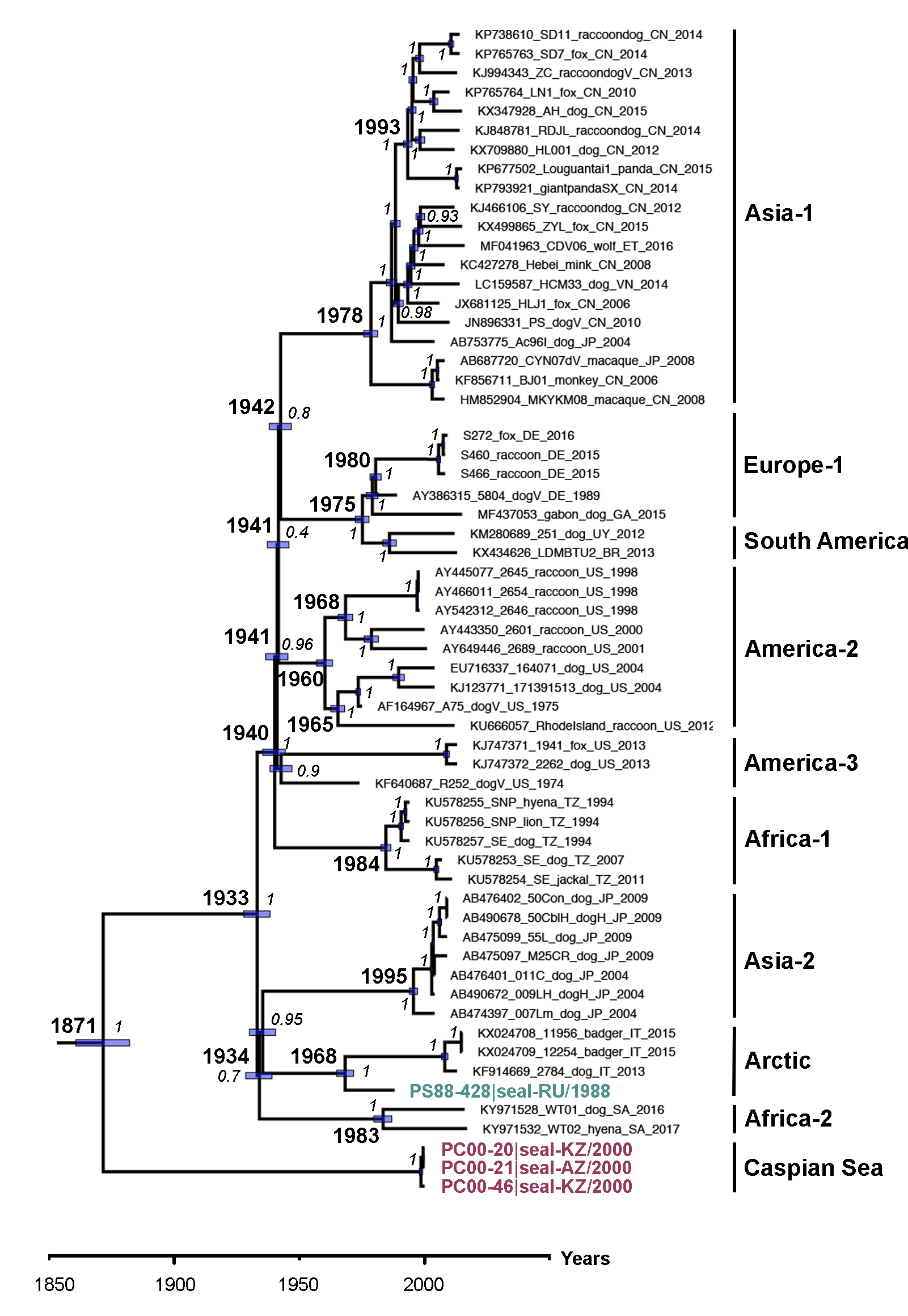

Supplement: Supplementary file 1 [file viruses-11-00894-s001.zip › Fig-S3.jpg]

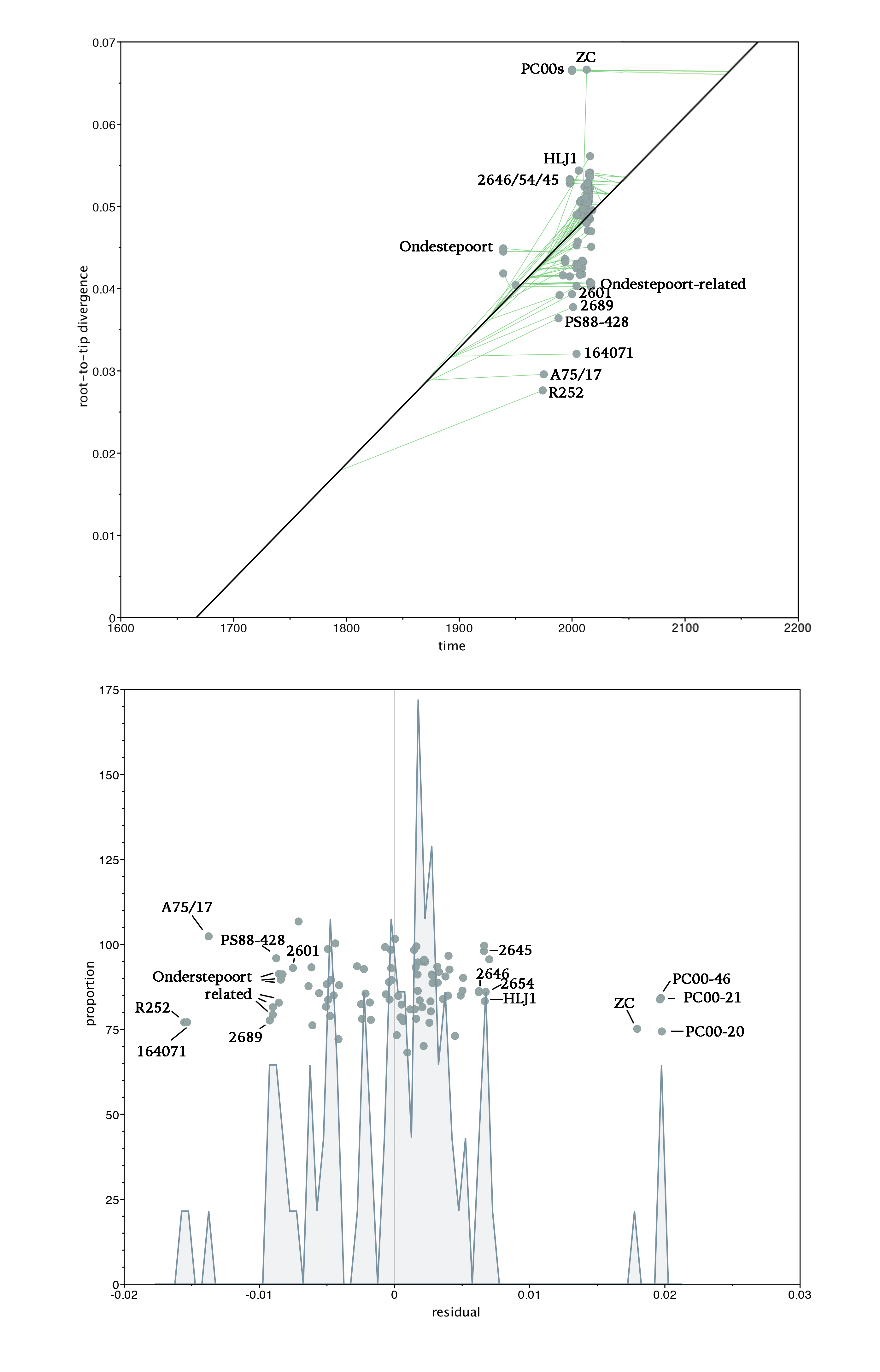

Supplement: Supplementary file 1 [file viruses-11-00894-s001.zip › Fig-S4.jpg]

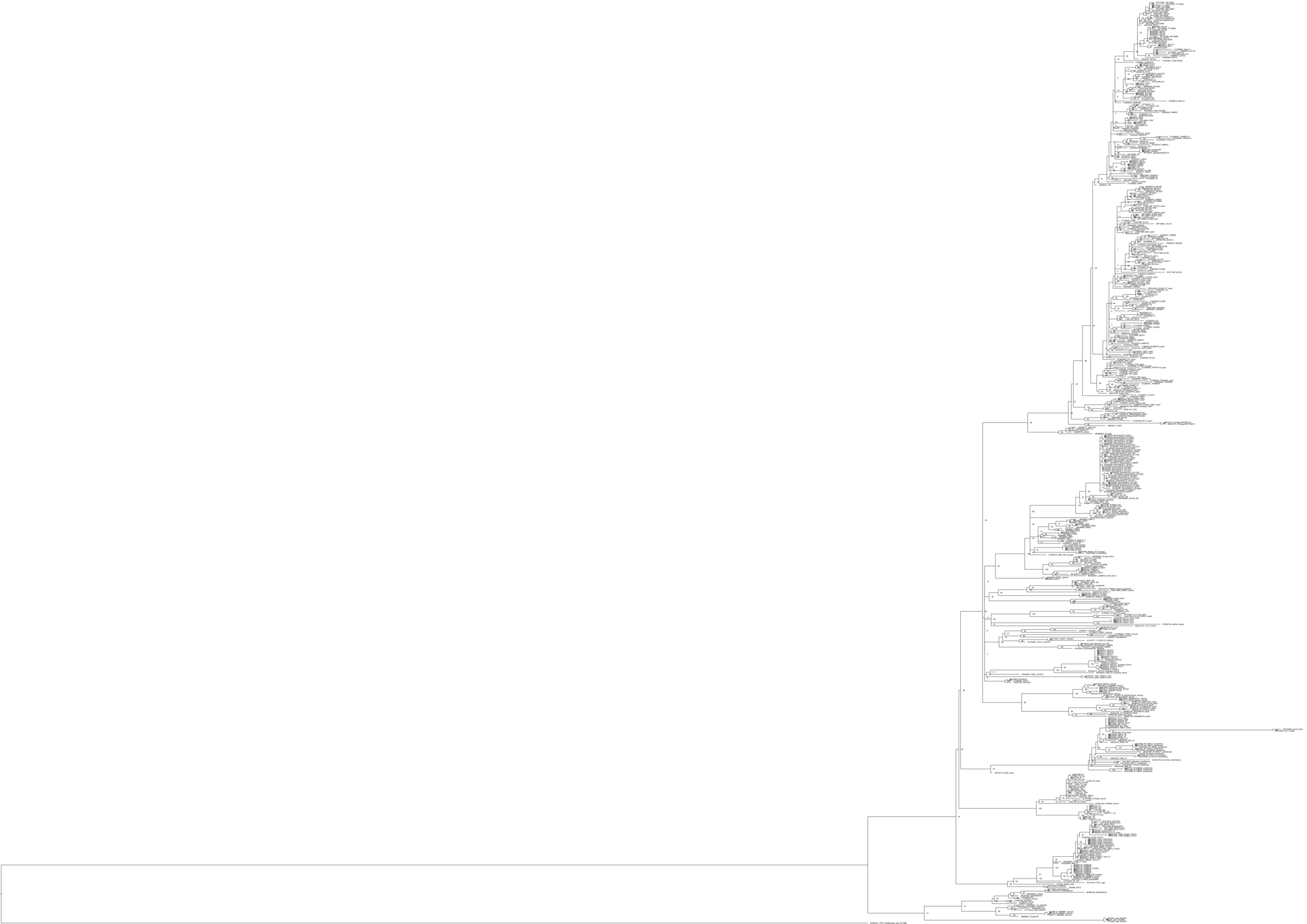

Supplement: Supplementary file 1 [file viruses-11-00894-s001.zip › FigS5.pdf]
